# Bayesian inference from single spikes

**DOI:** 10.1186/1471-2202-14-S1-P278

**Published:** 2013-07-08

**Authors:** Travis Monk, Michael Paulin

**Affiliations:** 1 Department of Zoology, University of Otago, Dunedin, New Zealand

## 

Spiking neurons appear to have evolved concurrently with the advent of animal-on-animal predation, near the onset of the Cambrian explosion 543 million years ago. We hypothesize that strong selection pressures of predator-prey interactions can explain the evolution of spiking neurons. The fossil record and molecular phylogeny indicate that animals existed without neurons for at least 100 million years prior to the Cambrian explosion. The first animals with nervous systems may have been derived sponge larvae that started feeding in the water column [1].

We use models and computer simulations of predator-prey interactions to show that thresholding prey proximity detectors can greatly improve a predator's performance under certain ecological conditions. If a prey produces a stimulus, then there is a critical stimulus level at which a predator's expected energetic return for striking exceeds the expected return for not striking. A predator with a mechanism for triggering a strike when the stimulus reaches this critical level has a massive advantage over predators lacking such a mechanism. We suggest that the first neurons were threshold-detecting devices that served this function.

**Figure 1:**
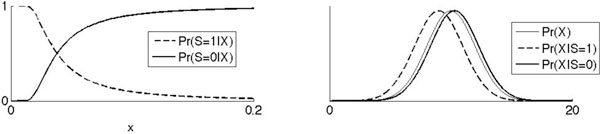
**Simulation of prey detection by spiking sensors**. The state *x *is the distance from predator to prey on a time step. The predator has a sensor that fires spikes with an intensity that depends on the strength of some stimulus produced by the prey; when *x *is small, the predator's sensor fires with higher intensity (and vice versa, left subplot). In general, we can approximate this intensity from the biophysics of some real sensor and stimulus, but for simplicity we assume that the intensity falls as $1/x^{2}$. Pr(S = 1|X) and Pr(S = 0|X) are the probabilities of the sensor firing in some small window of time given *x*, and we consider sufficiently small time windows such that no more than one spike can occur in that window. Given these conditional pdfs, it is possible to infer prey location using the sensor's spikes by Bayes' rule (right subplot). The prior distribution of *x *is Pr(X). We can then calculate the posterior distribution of *x *given the output of a spiking sensor, Pr(X|S = 1) and Pr(X|S = 0). On the next time step, this posterior becomes the new prior and the process repeats. When spikes and non-spikes are streamed very quickly, these posterior pdfs are updated almost continuously and in real-time.

According to our model, neurons evolved as proximity detectors. We show that although these spiking detectors evolved to maximize the rate of prey capture, it is possible to use spikes to determine the location of prey by Bayesian inference. We show that inferring prey location from individual spikes has higher utility than using inter-spike intervals or rates. The conditional probability density function (pdf) of prey location given the output of such a detector necessarily has smaller entropy on average than the marginal pdf of prey location (Figure 1). Therefore, a single spike (or non-spike) from such a neuron can be interpreted not only as an assertion about the presence of prey in the vicinity, or as a command to strike, but also as an assertion about the location of the prey at that time. It follows that individual spikes from threshold-detectors can, in principle, be used to infer prey location by Bayes' rule [2] as shown in Figure 1. Bayes' rule is the best strategy to modify beliefs based on imperfect evidence or data, and any animal that evolved the capacity to infer prey location from streaming neuronal spikes would have outperformed its competitors.
